# Designing of an extract production protocol for industrial application of cell‐free protein synthesis technology: Building from a current best practice to a quality by design approach

**DOI:** 10.1049/enb2.12029

**Published:** 2023-12-06

**Authors:** Beatrice Judith Melinek, Jade Tuck, Philip Probert, Harvey Branton, Daniel G. Bracewell

**Affiliations:** ^1^ Department of Biochemical Engineering UCL London UK; ^2^ CPI Darlington UK; ^3^ Merck KGaA Darmstadt Germany; ^4^ eXmoor Pharma Concepts Ltd Darlington UK

**Keywords:** biochemical engineering, bioprocess engineering, characterisation, optimisation, standardisation

## Abstract

Cell‐Free Protein Synthesis (CFPS) has, over the past decade, seen a substantial increase in interest from both academia and industry. Applications range from fundamental research, through high‐throughput screening to niche manufacture of therapeutic products. This review/perspective focuses on Quality Control in CFPS. The importance and difficulty of measuring the Raw Material Attributes (RMAs) of whole cell extract, such as constituent protein and metabolite concentrations, and of understanding and controlling these complicated enzymatic reactions is explored, for both centralised and distributed industrial production of biotherapeutics. It is suggested that a robust cell‐free extract production process should produce cell extract of consistent quality; however, demonstrating this is challenging without a full understanding of the RMAs and their interaction with reaction conditions and product. Lack of technology transfer and knowledge sharing is identified as a key limiting factor in the development of CFPS. The article draws upon the experiences of industrial process specialists, discussions within the Future Targeted Healthcare Manufacturing Hub Specialist Working Groups and evidence drawn from various sources to identify sources of process variation and to propose an initial guide towards systematisation of CFPS process development and reporting. These proposals include the development of small scale screening tools, consistent reporting of selected process parameters and analytics and application of industrial thinking and manufacturability to protocol development.

## Graphical abstract

Cell‐Free Protein Synthesis (CFPS) has, over the past decade, seen a substantial increase in interest from both academia and industry. This review/perspective focuses on Quality Control in CFPS. The articles draws upon the experiences of industrial process specialists, discussions within the Future Targeted Healthcare Manufacturing Hub Specialist Working Groups and evidence drawn from various sources to identify sources of process variation and to propose an initial guide towards systematisation of CFPS process development and reporting.

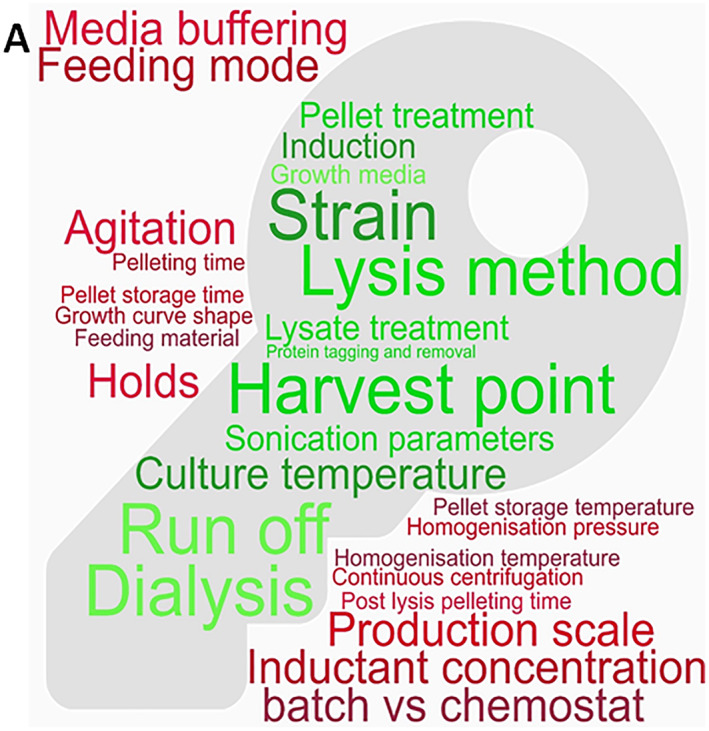

## 1 INTRODUCTION

### 1.1 Introduction to CFPS

The use of Cell‐Free Protein Synthesis (CFPS) also known as in vitro transcription and translation (IVTT) for the production of a protein (phenylalanine) was first published in 1961 [1]. CFPS subsequently played a fundamental role in helping to understand cellular processes and genetics. It has since been used to produce a variety of therapeutic proteins including antibodies, vaccine candidates and protein biologics [2] as well as for optimising metabolic pathways and biomolecule design [3].

Therapeutic proteins are an important and growing class of complex, specific and highly effective medicines. The vast majority of therapeutic proteins are currently produced using recombinant DNA technology in a range of production systems from bacteria such as *Escherichia coli*, through eukaryotes such as CHO, to transgenic animals and plants [4]. All of these systems rely on the machinery contained in all living cells that allows them to make the proteins they need. In CFPS, the aforementioned machinery is employed in the absence of living cells for the transcription and translation of a DNA sequence and is extracted from living cells in the form of either a crude lysate or as purified recombinant elements. A living cell culture or organism needs to be simultaneously maintained and, at the appropriate juncture, induced to make the product of interest. This process takes days, if not weeks, to complete. CFPS, by contrast, involves a set of chemical reactions, which can be initiated at will from frozen or lyophilised reagents (extract, a reaction mix to supply building blocks and energy source molecules, and a DNA template) and can produce useful amounts of protein within hours. This flexibility has stimulated interest in CFPS as a potential platform for the on‐demand manufacture of therapeutic proteins [2, 5, 6, 7].

### 1.2 Commercial application of CFPS

The use of CFPS in production is currently limited to a handful of examples. Sutro Biopharma is at present the leader in the field, with a number of antibody drug conjugates produced by CFPS in early‐phase clinical trials. Vaxcyte (formally Sutro Vax) has a number of mainly conjugate vaccine candidates in preclinical development. They focus on the greater control of conjugation sites and number afforded by the use of non‐natural amino acids [8]. They have attracted substantial funding and interest from large pharmaceutical partners. Another often quoted application for CFPS is in toxic product manufacture [9], where the product may be toxic to the producing cells or may represent a biohazard to the production personnel. Ipsen Biopharma, for example, who produce the highly potent, but highly toxic therapeutic botulinum, recently began exploring the use of CFPS in collaboration with the UK‐based CPI and Touchlight Genetics and with funding from Innovate UK [10] (CPI is the Centre for Process Innovation, a social enterprise and innovation catalyst, originally established by a UK government agency).

Expanding the definition of Cell‐Free Synthesis to include the production of nucleic acids, such as messenger RNA (mRNA) and plasmid DNA (pDNA), there is growing interest in both fields. Prior to the COVID‐19 pandemic, the commercial application of mRNA was in pesticides and plant and fish vaccines. Greenlight Biosciences is an example of a key player in this field. Although much of the technological knowhow was already in place, therefore, the production and in‐human administration of mRNA in particular have been accelerated by the COVID‐19 pandemic with production by Pfizer/BioNtech, Moderna and Curevac to name only a few [11, 12]. There are a growing number of start‐ups in cell‐free pDNA production including, but not limited to, Touchlight and 4basebio, where a key advantage is the possibility of excluding bacterial sequences, particularly antibiotic resistance genes [13]. The enzymatic reactions used to make nucleic acids are, however, relatively simple when compared to CFPS.

### 1.3 Regulatory considerations

The limited commercial use of CFPS means that there is no regulatory precedent to follow, and quality control approaches remain to be fully elucidated. Concerns around maintaining reagent [14, 15, 16] and reaction consistency in particular will need to be addressed. There is evidence to suggest that with automation, better process and environmental controls and process understanding derived from Multivariate Data Analysis (MVDA) [17, 18] reaction‐related variations can be minimised [19, 20, 21, 22]. Further, some of the reagent‐related variability can be mitigated, for example, by modifying buffer magnesium salt levels [23, 24]. However, the reagents are complex, particularly the extract, which is a mixture of numerous enzymes, the concentration and activity of which may vary according to the extract preparation (culture and processing). While in theory, we would not expect any dramatic difference in a cell extract activity, assuming a consistent preparation method, from batch to batch [14], in practice, this is either not the case or remains to be demonstrated definitively in a product‐independent manner.

### 1.4 Scope of article

The authors from UCL are researchers on the Future Targeted Healthcare Manufacturing (FTHM) Hub, whose interest in CFPS stems from its potential use as a platform for stratified medicines manufacture [2]. The CPI‐based authors were key members of the team producing cell extract for the aforementioned collaborative project between Ipsen, CPI and Touchlight, and industrial users of the FTHM Hub. Extensive discussions within the FTHM Hub were recently summarised in an article for BPI [10], with significant concern amongst industrialists regarding how CFPS consistency can be assured and measured to regulatory standards. It is this question we aim to explore further here.

In this paper, we first describe a pragmatic approach to tackling this problem, with specific reference to the experience and findings of CPI, who have developed their own in‐house method for industrial scale extract production. The pragmatic approach involves relying on the consistency of the process to give a consistent reagent (cell extract, plasmid DNA and polymerase) and relying on the easier manipulation of the CFPS reaction itself to adjust for any batch‐to‐batch variability. However, this pragmatic approach does not fully answer the question of how we might make an extract for CFPS reactions which can be used as a replicable plug‐and‐play platform process across multiple products and sites. Such a plug‐and‐play platform process can only be achieved through a fuller identification of *all* process parameters which significantly impact upon the properties of the extract and which themselves impact upon the subsequent reaction outcomes, irrespective of the protein being made. We therefore extended the analysis, based on the experience of the authors and data from a comprehensive literature review, by using a Quality by Design (QbD) approach to identify the most significant parameters to control and recommend characteristics of the extract/reaction for which analytics need to be incorporated, perfected or developed.

## 2 CPI CASE STUDY

Cell‐free Protein Synthesis (CFPS) faces challenges due to raw material variability, particularly in cell‐derived components like the cell‐free extract and DNA, causing inconsistent performance. Further, the complexity of these same components makes identifying the source of the performance inconsistencies difficult. In this section, we discuss a pragmatic approach, which can be applied when only a small number of products are being produced: developing a consistent and accessible cell‐free extract production process and then rapidly screening and adjusting reaction parameters in the CFPS reaction to account for any remaining batch‐to‐batch variability in the extract. This is based on the approach developed by the team at CPI and their experience.

### 2.1 Industrial production of a toxic therapeutic protein: Project background, approaches and rationale

CFPS provides a potential alternative manufacturing method that is not confined to the host strains physiological range and has improved biocontainment. These features are important in addressing the challenges around the production of proteins that are toxic to their host cells and/or production personnel, such as recombinant botulinum toxin. Innovate UK funded a project that brought together the companies CPI, Ipsen Biopharm and Touchlight Genetics to produce this highly potent product in a Good Manufacturing Practice compliant, industrial‐scale CFPS reaction.

Raw material variability is a challenge for CFPS. CFPS reactions are initiated by combining a reaction mix, enzyme mix, DNA template and, usually, an exogenous RNA polymerase. The reaction mix may come in a wide variety of formulations and contains energy source molecules and reaction building blocks. The enzyme mix (also known as cell‐free exact) contains enzymes for transcription, translation and energy source molecule recycling and may come in the form of a raw cell lysate from one of a variety of organisms, or be reconstituted from purified recombinant proteins. Many of these raw materials are cell‐derived, including the cell‐free extract and DNA. Batch–to‐batch variability in these cell‐derived components means a consistent CFPS performance is difficult to achieve.

Plasmid DNA is typically produced by *E. coli* cultures, but small differences in plasmid quality arising from cell culture and purification conditions result in large differences in protein yield from CFPS reactions [25, 26, 27]. Touchlight's proprietary linear DNA vector, doggybone DNA (dbDNA^TM^), which is produced synthetically, was used as the reaction template in this project. The aim of this was to ensure that there was greater consistency in the quality of template DNA. In theory, as the template is synthetically produced, there should be less biological variability. Due to the limited number of batches required for this study, demonstrating this consistency was not included in the scope of the work.

The CPI's team role in this project was the development and implementation of the Cell‐free extract production and CFPS reaction protocols. This will therefore be the focus of the discussion in this paper. Early in the project, a robust, scalable and transferable extract production process was identified as key. Indeed, ideally, to allow for the wide use of this technology, the extract production should be possible even by non‐expert manufacturers.

Identification of critical Raw Material Attributes (RMAs) is important to ensure the measurable consistency of the cell‐free extract. Many of the RMAs which are known to be important, such as activity and protein content, can be measured with relative ease using standard assays. However, other attributes which are known to be important, such as ribosomal integrity and concentration of inhibitory agents including proteases and nucleases, are difficult to measure. It was assumed therefore that a consistent production process, operating within a set tolerance, should produce a similarly consistent cell‐free extract. Further, with tight process control, the CFPS reaction itself should be sufficiently robust for product titre variation to be minimal for a given batch of the cell‐free extract. Further, if Critical Process Parameters in the CFPS reaction could be modulated, from one cell‐free extract batch to another, it was hypothesised that consistent performance could be achieved.

Over 36‐month, a scalable and robust cell‐free extract manufacturing process was generated, high‐throughput screening tools were applied to rapidly assess CFPS reaction conditions and a high‐performing and scalable CFPS protocol was developed.

### 2.2 Achieving a consistent lysate generation process

The approach taken was to first define a cell‐free extract generation process that was free of unnecessary lysate processing steps, whilst being scalable and transferable, with selected quality parameters measured to support consistent material generation.

**FIGURE 1:**
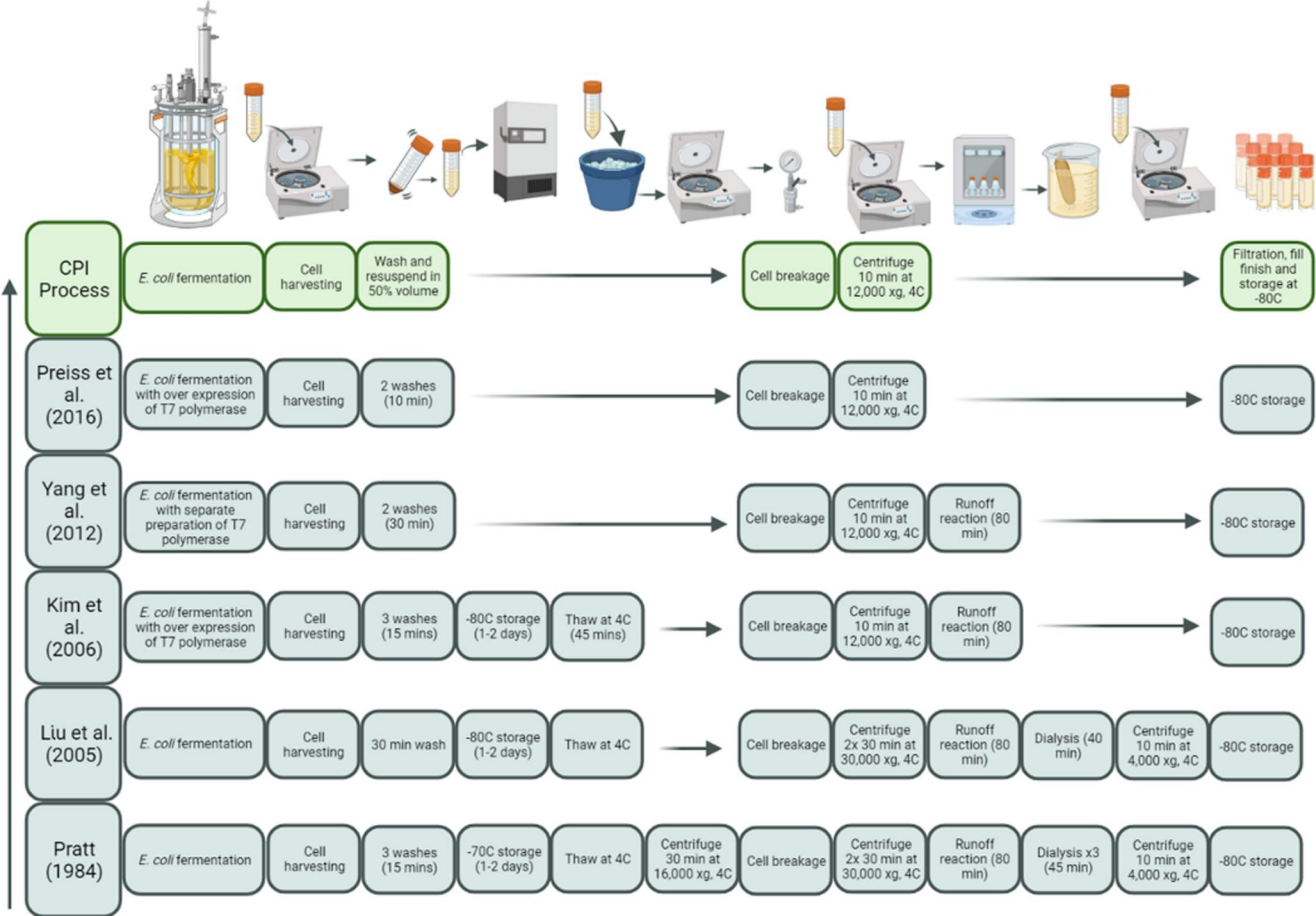
Evolution of the extract generation process.

Laboratory scale cell‐free extract generation processes for CFPS are widely described in the literature. Many of these are long, multi‐step processes which give rise to manufacturing complexity and opportunities for inadvertent processing differences [28]. The aim therefore was to remove as many processing steps as possible to minimise the number of steps where variation could be introduced and create a robust process. Figure 1 shows the main steps in the current and historic processes. In post‐cell culture, it had been reported that two washes should be sufficient to remove contaminants contained in the culture medium [29]. Run‐off reactions are carried out after cell breakage and are aimed at removing endogenous mRNAs in the lysate. Run‐off reactions are often carried out using buffer systems which are not suitable for the subsequent CFPS reaction, so buffer exchange is required. The run‐off and dialysis steps involve long incubation periods during which the opportunity for the degradation of molecular machinery in the extract is increased potentially by reducing CFPS yields. The pellet wash, run‐off and dialysis steps were therefore identified as process steps, which it would be beneficial to remove.

An *E*. *coli* derived cell‐free extract using the BL21 Star^TM^ (DE3) (Thermo fisher) strain was used due to its thorough description, prevalence in industry and abundance of previous work [30, 31]. This strain has a mutation in the RNaseE gene that is involved in mRNA degradation, resulting in greater transcript stability.

Firstly, BL21 Star^TM^ *E. coli* was grown in a selection of medium in shake flasks; cells were harvested, sonicated (due to low volumes) and tested for levels of expression in CFPS reactions. Notably, lysate generated from biomass grown with minimal media showed no activity in CFPS reactions and had significantly lower protein concentrations (Supporting Information S1).

Conditions including growth media, temperature and harvest criteria, which showed best expression, were scaled to ∼100 mL in an advanced micro‐scale bioreactor (ambr250^®^). Additional process parameters such as pH and partial pressure of oxygen (pO_2_), which could not be controlled at shake flask scale, were optimised. Typical process parameters such as OD_600_ (optical density at 600 nm, a measure of cell concentration), temperature, pH, dissolved oxygen (DO) and carbon evolution rate (CER) were recorded and used to scale to 10L. A chill step is required to arrest growth, such that cells are harvested in the exponential phase, when cells are most metabolically active, to achieve a high yielding cell‐free extract. Trigger OD_600_ values were investigated, with initial results suggesting that initiating chilling at OD_600_ = 12 with a 2 h chill would give a final OD_600_ = 23, which was within the exponential phase. Using the aforementioned parameters, the process was scaled to 10L. The initial run demonstrated that the cooling strategy was not sufficiently aggressive to arrest growth at larger scale, as the bulk of the culture was not cooled quickly enough, and therefore the trigger point was adjusted to OD_600_ = 14 with a 1 h chill.

**FIGURE 2:**
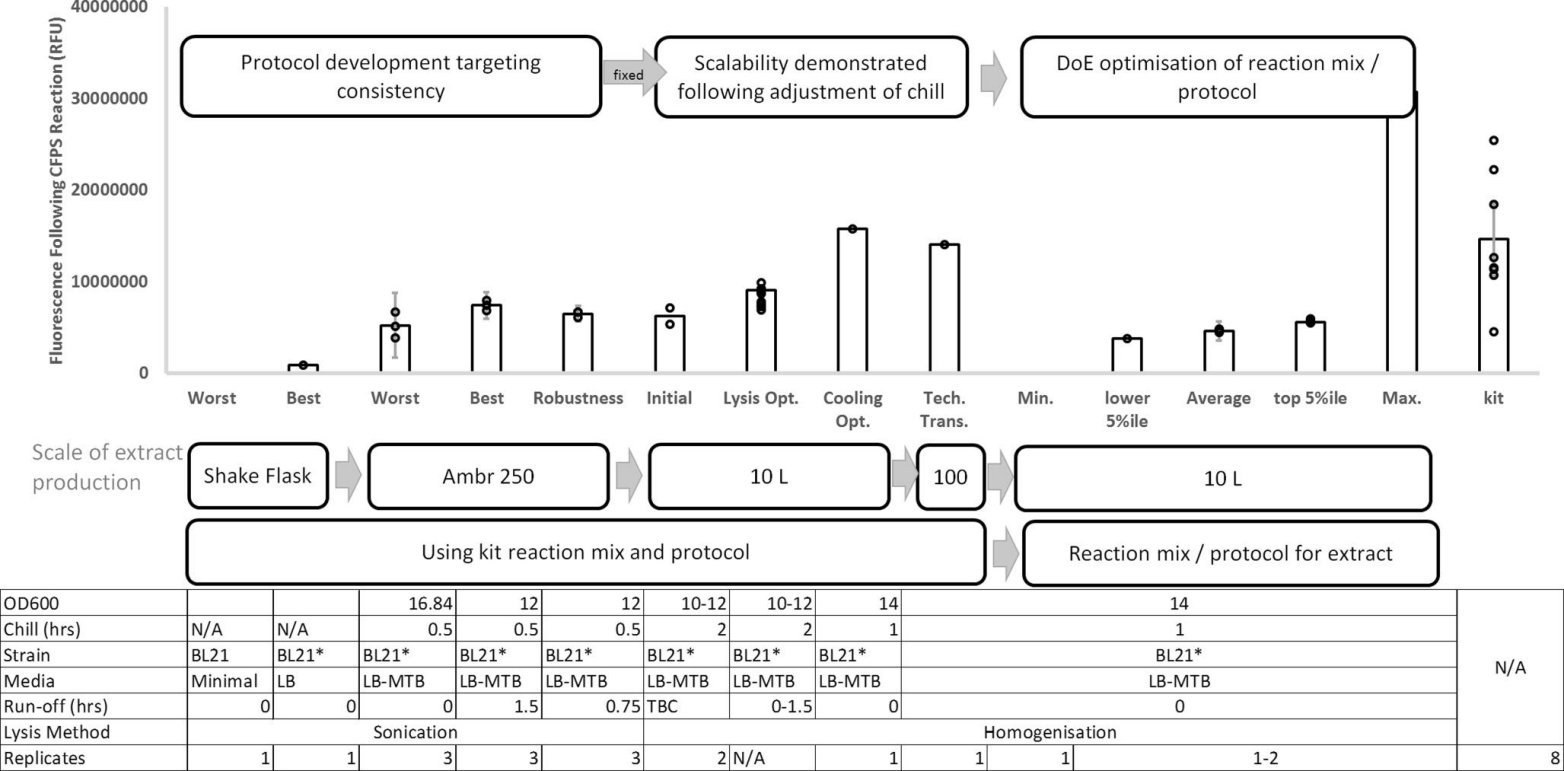
Overview of the process used by CPI to develop their cell‐free extract production protocol.

Using representative material generated at 10L scale, post‐harvest lysate processing steps were tested. Previous work was carried out using sonication as the method of cell breakage; however, at these larger volumes, the cell breakage method was changed to high‐pressure homogenisation to enable greater throughput and industrial relevance. Two homogenisation pressures were tested (850 and 950 bar), with either two or three passes with subsequent incubation for up to 90 min to establish the impact of the run‐off reaction. Next, lysate was clarified by centrifugation, 0.45 μm filtered and stored at −80°C until CFPS reactions were carried out. Figure 2 demonstrates that lysate performance for CFPS was robust to different homogenisation conditions. Therefore, 850 bar with three passes was selected as these conditions showed the most robustness to differences in cell culture conditions. Run‐off reactions did not appear to change the activity of the lysate, and therefore it was decided that this processing step would be removed in an attempt to create a leaner process, with fewer steps to accumulate variation. The experience of chill strategy efficacy between scales indicated that further work on these parameters may be required. The impact of chill parameters (trigger OD_600_ and rate) and selected post‐harvest processing steps was therefore further assessed at 10L scale. Following several assessment runs to demonstrate robustness, the process was transferred and scaled to 100L by a Contract Manufacturing Organisation (Figure 2).

The overall process development scheme and impact of optimisation at each stage are represented in Figure 2.

### 2.3 Use of design of experiments to optimise the CFPS for a specific lysate

The initial intention was to carry out the screening of a range of factors such as energy sources, including creatine kinase, pyruvate, glucose and starch, in addition to screening chemical additives and biochemicals. It quickly became apparent that this was expensive, laborious and the reaction set‐up was prone to error. The linear scalability of CFPS reactions enabled optimisation work to be completed at microscale in a plate format, which could be prepared by a liquid handling robot, before scaling up to larger volumes. To enable fast screening and development of a screening platform, an enhanced green fluorescent protein (eGFP) construct was chosen so that protein expression could be monitored easily in real‐time. Initial assessments demonstrated the importance of good mixing and sensitivity to minor deviations in the reaction set‐up. Therefore, CFPS scale was increased to 100 μL, and mixing and temperature control were automated using a microplate reader.

Design of Experiments approaches were applied to the optimisation of the CFPS reaction using lysate generated in the 100L batch. The use of lysate from the same batch allowed comparability between screens, without introducing the possibility of lysate batch‐to‐batch variability. Screens were planned with Design Expert software using a similar approach to conventional process development; however, more conditions, than typically would be explored, were possible due to a microplate format. Despite this, typical process parameters such as pH, temperature and dissolved oxygen (pO_2_) could not be controlled although plates with sensors and fluorescent dyes were used to monitor conditions to enable further processing information to be gathered.

A range of responses were observed which highlights the possibility of potential improvements. All reactions plateaued after a relatively short time, a feature of CFPS commonly attributed to the accumulation of inhibitory by‐products or the depletion of critical components. The impact of feeding after the reaction plateau with key reagents such as magnesium and nucleoside triphosphates (NTPs) was therefore also assessed [32]. Subsequent screens were designed iteratively, and the design space to be explored was informed by the previous screen, enabling sufficient optimisation. The high‐level impact of this optimisation can be seen in Figure 2.

After optimal reaction conditions were identified, the process was scaled firstly to 1 mL scale in a 24‐well plate format using an Applikon micro‐Matrix bioreactor system. This allowed for the control of agitation, temperature, pO_2_ and pH as well as testing process robustness. Next, the process was scaled to 6 mL scale in the Sartorius advanced micro‐scale bioreactor (ambr15^®^) system which allowed for the exploration of agitation in a more representative manner and therefore the relationship with pO_2_. Additionally, although it may seem trivial, reaction start conditions such as temperature equilibration of mixes to avoid temperature ramps and unexpected enzyme activity were considered. It was found that good pO_2_ and pH control were pertinent to good yield and therefore PID (proportional‐integral‐derivative) controller settings, which on utilised systems are by default configured for cell‐based processes, were modified to significantly improve the speed of response as required by shorter cell‐free reactions.

**FIGURE 3:**
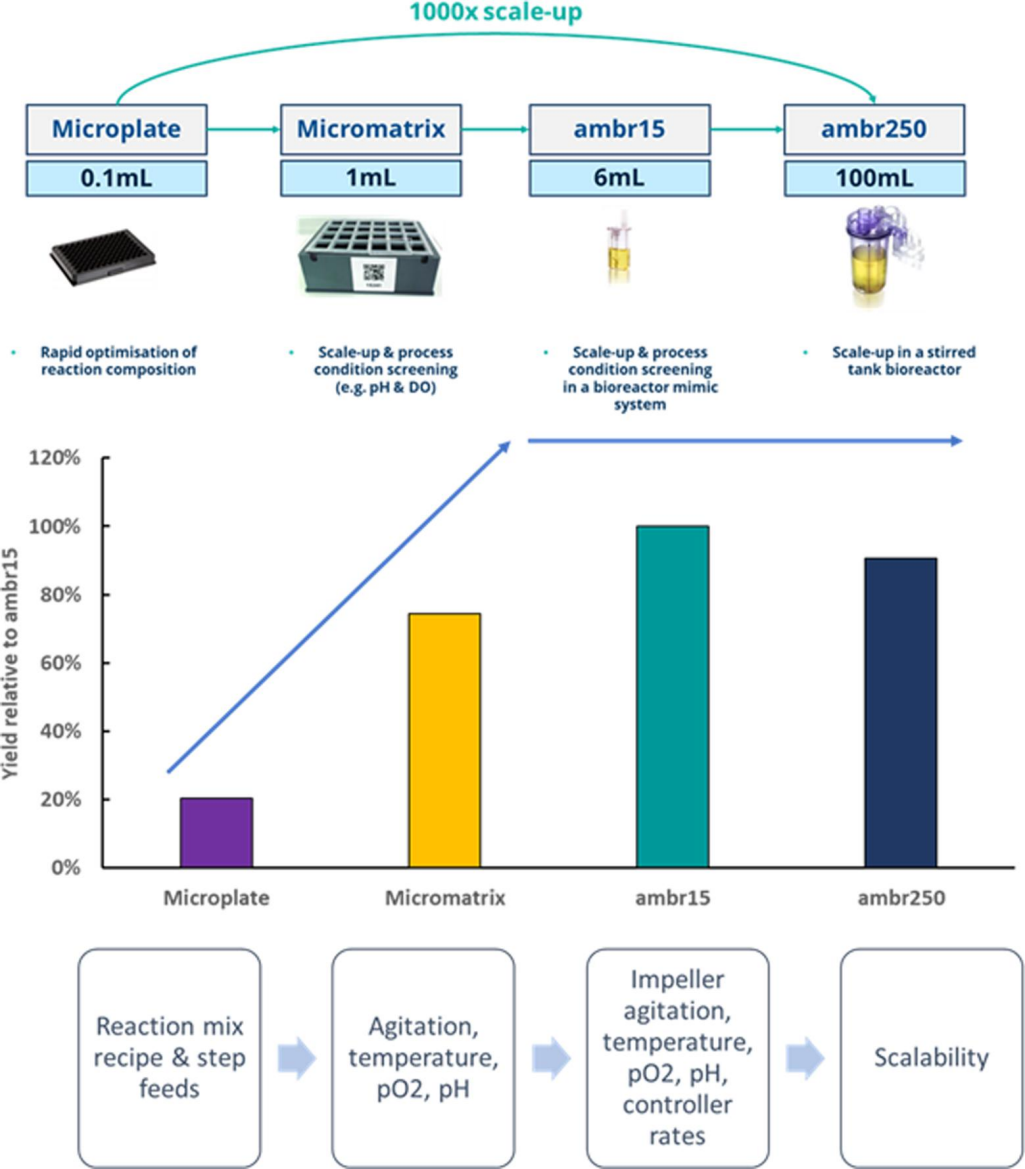
Schematic summary of the scale‐up journey for the process development of the CFPS reaction as conducted by CPI.

Data showed that over a 1000‐fold increase in scale, improvement in yield and process robustness were observed (Figure 3). Improved yields could be attributed to a more accurate CFPS reaction set‐up and better process control highlighting their importance to support effective CFPS.

## 3 CFPS AS A REPLICABLE PLUG‐AND‐PLAY PLATFORM

Cell‐Free Protein Synthesis offers advantages over cell‐based production, including rapid development and potential for automated, stand‐alone devices. It therefore has the potential to be a versatile platform for distributed manufacturing. Specifically, centralised extract production and controlled CFPS reactions at local sites can enable a practical implementation of personalised medicines. This would require monitoring extract variability and its impact on the production of different protein types, rapidly optimising CFPS reactions, and establishing sufficiently consistent extract properties. This section focuses on identifying key process parameters impacting CFPS product yield and addresses the need for Quality by Design (QbD) approaches for a plug‐and‐play platform process. A systematic analysis of parameters impacting extract production and CFPS reactions is presented, emphasising at this stage the importance of product yield and robustness, as pre‐requisites for the further development of the technology.

### 3.1 Distributed manufacture using CFPS

#### 3.1.1 The potential and the barriers

The previous section describes a holistic approach, which exemplifies current best practice for the development of a commercial manufacturing process using CFPS. The process described is for a high‐value product with specific features, in this case, high biosafety risks, for which CFPS presents a unique solution. However, CFPS has potential as a platform process, with a number of key advantages over existing cell‐based production systems including rapid, high‐throughput process development and potential for production in a stand‐alone automated device [10].

It is possible to imagine a scenario where the cell‐free extract production is centralised and controlled, and CFPS reactions are employed for localised small‐scale production. Such a scenario would be a significant enabler for the practical implementation of stratified and personalised medicines.

With a centralised extract production, the production process and extract properties could be closely monitored and controlled. Further, additional strategies could be implemented such as pre‐purification to remove process‐related impurities, including endotoxin, and the presence of any adventitious organisms, can be measured and controlled. The final product purification process could also be designed to ensure a consistent removal of undesired process‐related impurities to below acceptable concentrations, given a known and consistent background.

The CFPS reaction for each product type could also be developed and optimised centrally, with the process conditions programmed into an automated production device. Thus the possibility for rapid and high‐throughput process development could translate directly into a fast turn‐around from product development to production, with minimal risks of failure from technology transfer.

This would allow CFPS's potential for improved robustness and flexibility to be exploited. In particular, CFPS would allow multiple, small‐scale and simple to run production processes, with minimal infrastructure requirements. Such processes could be present in clinical settings, producing the multiple and various therapeutics that may be required.

However, CFPS is a complicated and, as yet, not fully understood technology. The above vision cannot be realised without addressing the complexity and characterisation of the raw materials (particularly the cell extract), improving the understanding of which process parameters are critical and the development of an in‐process monitoring strategy [10].

#### 3.1.2 Section methodology overview: Literature review and understanding the current state of the technology as a guide to future improvements

This section seeks to begin to answer the question of how the process understanding of CFPS can be systematically improved, allowing, in the longer term, for Quality by Design (QbD) approaches to be applied, further streamline process development, and ultimately to generate a plug‐and‐play platform process. Guided by the remit of the Future Targeted Healthcare Manufacturing (FTHM) Hub, the ultimate goal is to establish guidelines for the development of an automated distributed manufacturing system.

**FIGURE 4:**
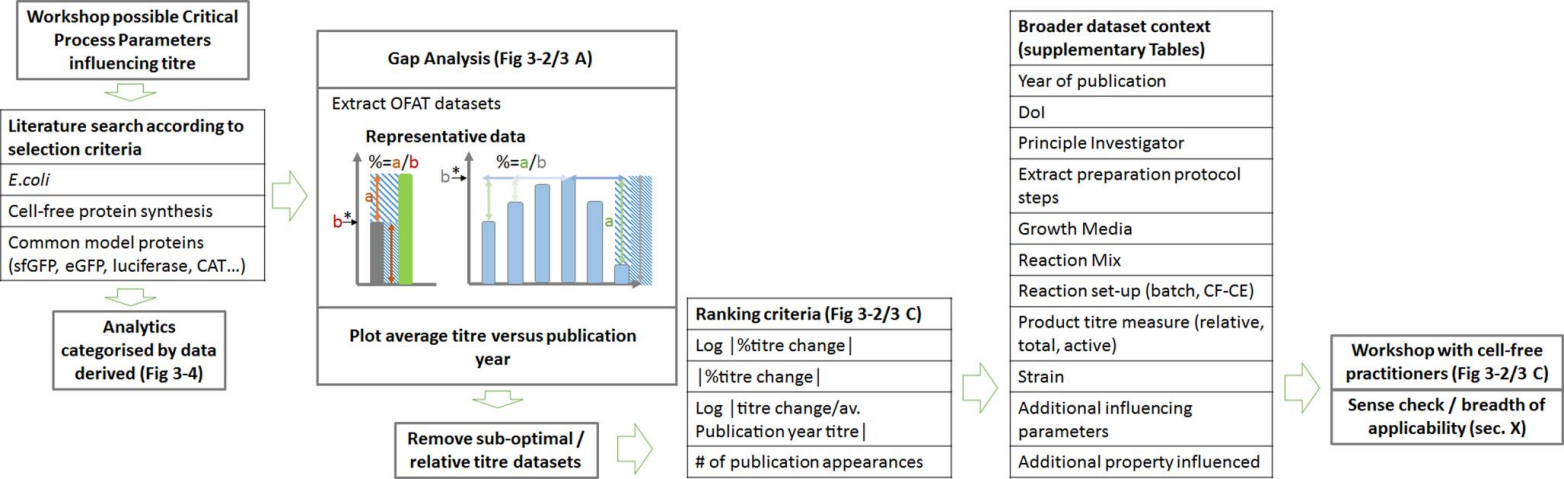
Illustration of the approach to the literature search, data extracted from the literature search, and methodology used to derive a top 10 possible Critical Process Parameter ranking for titre based on data extracted from the literature.

The authors aimed to do this by identifying and ranking parameters which have been shown in the literature to have an impact on CFPS product titre, which will impact active product dosage and downstream performance. The approach to this literature search and analysis is summarised in Figure 4. It is proposed that by focusing the process development and analytics on the most impactful process parameters, variability can be reduced and the robustness required for an automated CFPS system assured. These process parameters are also used to infer analytical and control strategies that may allow real‐time release testing.

**FIGURE 5:**
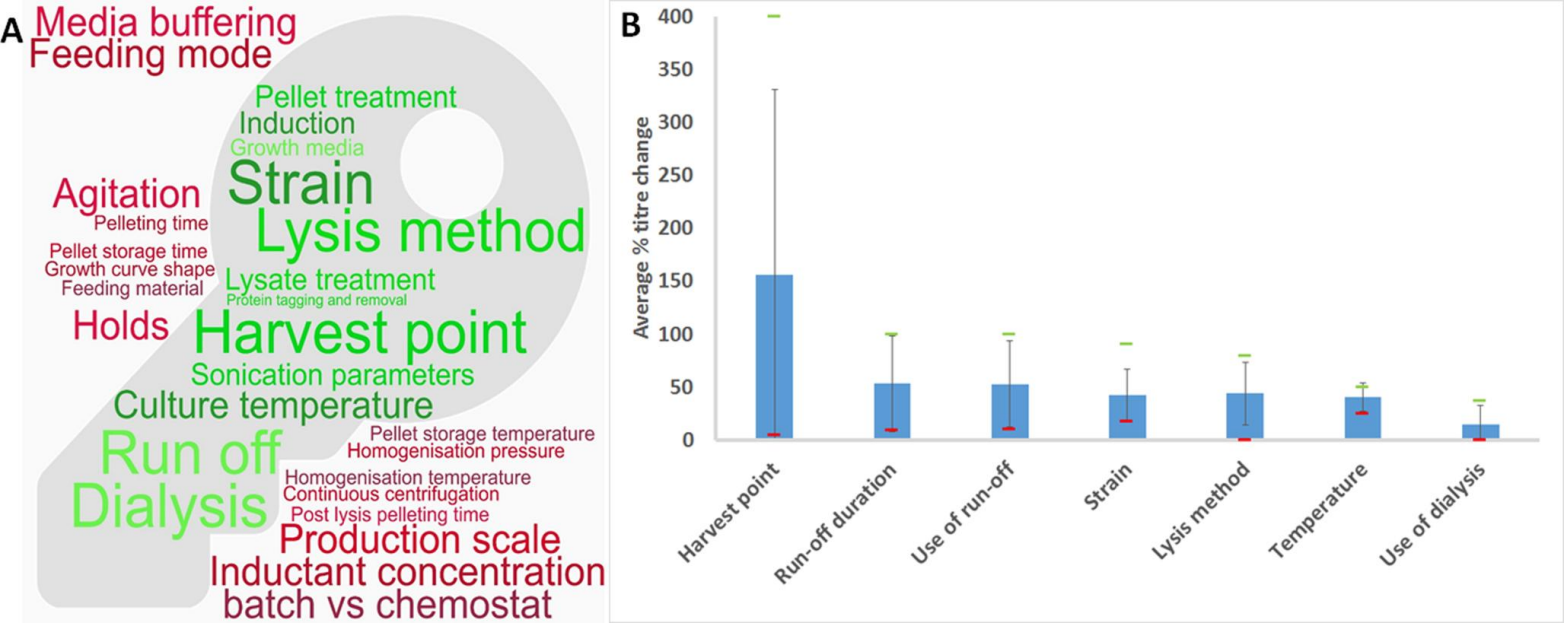
Summary of evaluation for possible Critical Process Parameters (possible CPPs) for the extract production process. (a) Word cloud, showing possible CPPs, identified by the authors during a workshop. Possible CPPs for which data is available in the literature are shown inside the key icon in green; those outside in red have not been found in the literature. For possible CPPs available in the literature, the volume occupied by the word (i.e., text size) is proportional to the number of appearances in the literature. For the ease of legibility, some of these possible CPPs have been amalgamated into broad categories; please see supplementary materials for a fuller breakdown of possible CPPs. (b) Bar charts, showing the average impact on titre of the possible CPPs for which more than three research articles have been published. For each research article, an average representative value was derived, and this average was used to give the height of the bars; the green marker represents the maximum reported and the red markers the minimum for each possible CPP; error bars are standard deviations (n ≥ 3).

**FIGURE 6:**
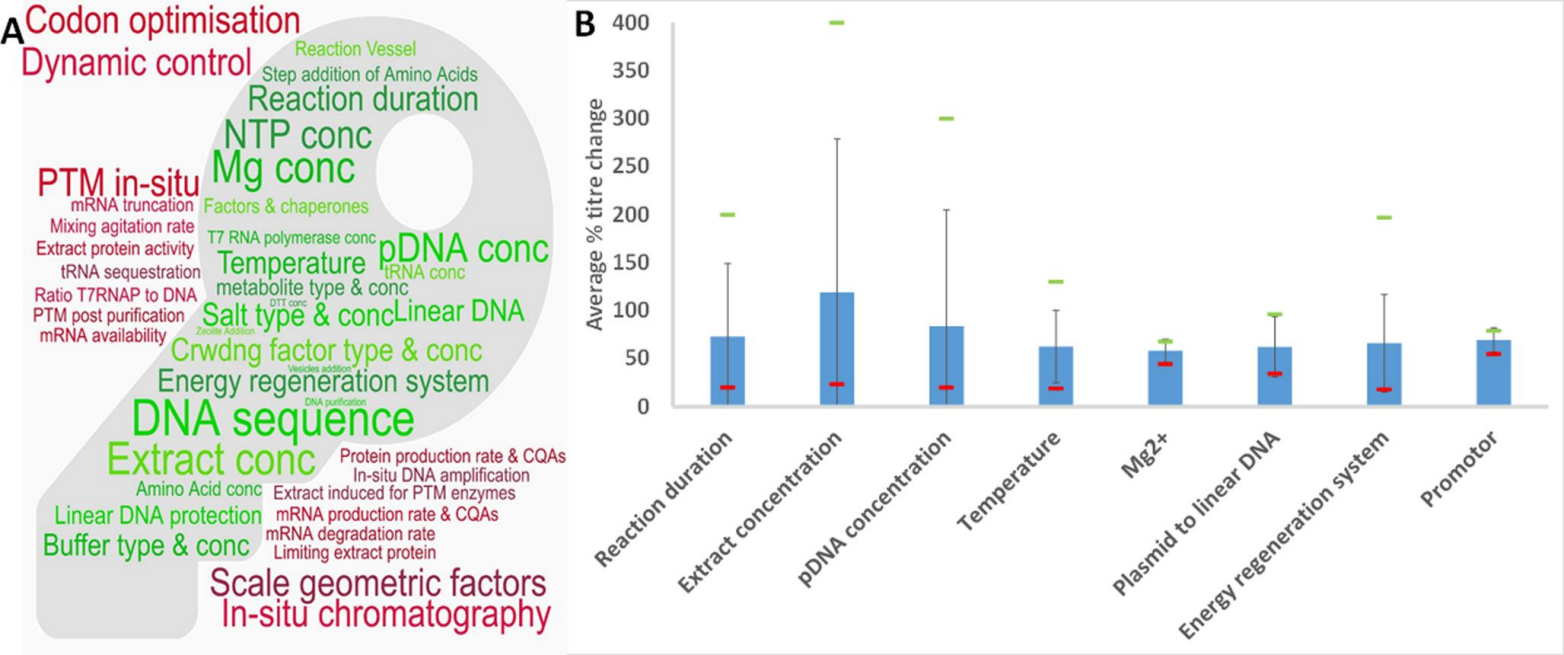
Summary of evaluation for possible Critical Process Parameters (possible CPPs) for the Cell‐Free Protein Synthesis Reaction. (a) Word cloud of possible CPPs. (b) Bar chart of average impact on titre of possible CPPs for which the number of research articles is greater than 3. (c) Top 10 ranking of possible CPPs impacting titre. For a detailed description of (a) and (b) see Figure 5 heading.

Possible approaches to the evaluation of extract Raw Material Attributes (RMAs) is explored later in the section. As the attributes of these cell‐free extracts are expected to be of great significance to the product Critical Quality Attributes (CQAs), and as the production of the extract can clearly be demarcated from the CFPS reaction itself, the extract production process and CFPS reaction process were evaluated separately (Figures 5 and 6).

**FIGURE 7:**
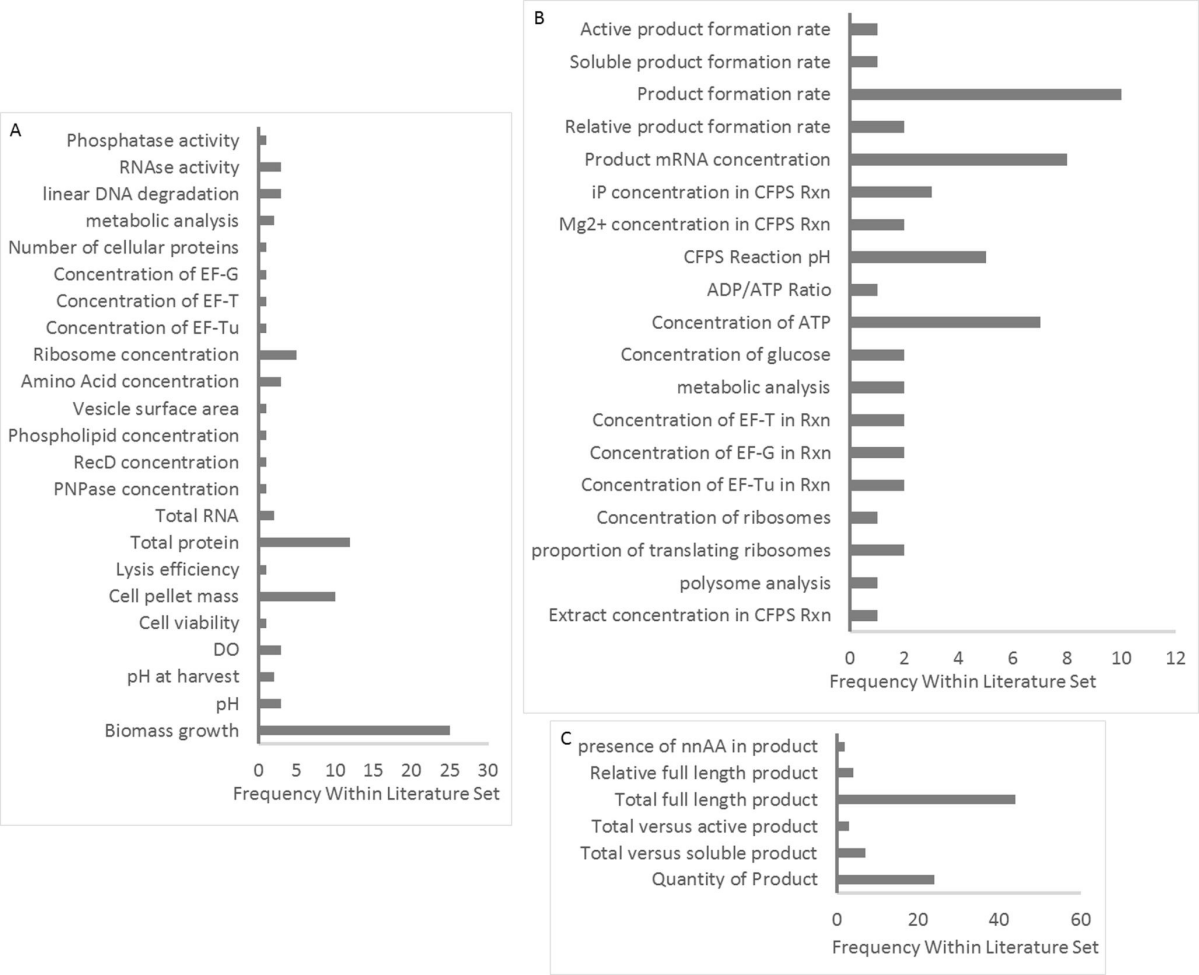
Parameters and qualities for which analysis has been conducted in the identified literature of which the total number was 58. Pertinent to (a) the extract production process and extract Raw Material Attributes, (b) monitoring of the Cell‐Free Protein Synthesis reaction and (c) evaluation of the final product Critical Quality Attributes.

Cell lysate attributes can be expected to impact directly upon the CQAs of the final active protein product. While some features of the extract are regularly measured, such as total protein (Figure 7a), no work has yet been published to link the full range of attributes of the extract to the product CQAs. Instead, most literature reports the effect of changes in the extract production process on the titre from the subsequent CFPS reaction. An evaluation to link RMAs to product CQAs should be easier for the PURE (Protein synthesis Using Recombinant Elements) extract, which may be considered analogous to a minimal media. However, as the whole cell lysate extract currently outperforms any PURE extract by 10× in terms of product titres, the focus of this article will be on whole cell lysates. For these reasons, the analysis in this paper aims to link the extract production process possible Critical Process Parameters (CPPs) to the CQAs of the resultant CFPS product.

Following an initial workshop, to identify possible CPPs from the authors' collective experience (Figures 5a and 6a), data was extracted, from an analysis of a range of literature, on the impact of single possible CPPs on titre (our chosen CQA). To further circumscribe the evaluation, data was collected for One Factor At a Time (OFAT) investigations, using *E*. *coli* cell extracts, for a limited range of model protein products (GFPs, CAT and luciferase). These model proteins were selected for their frequency in the literature and the relative ease and accuracy with which they can be quantified. *E*. *coli*‐derived lysate was selected also for its large body of literature, as the species with which the authors are familiar and as a suitable candidate for cost‐effective manufacture. Additionally, data was collected for each on the year of publication, baseline titre of the protocols used, research group, energy regeneration system, protein measurement (total, active, relative), reaction set‐up (batch, fed‐batch and continuous‐exchange), extract production steps, strain and growth media and other CQAs explicitly mentioned as effected and interactions with other possible CPPs identified by the study authors. This dataset has been made available in Supporting Information S1.

Tables 1 and 2 identify the top 10 possible CPPs based on the highest percentage change in titre from any one study. The average titre change over all the studies which included this parameter is also presented, along with the number of publications featuring this parameter, giving some indication, where a parameter has been widely studied, of the variability of impact levels recorded. This variability may imply that the importance of a given process parameter is dependent upon other process parameters or the process protocol. Studies with sub‐optimal and relative titre were excluded. This is in recognition that the percentage change will be weighted towards data points with lower initial starting or reference titres.

**TABLE 1:** Top 10 possible Critical Process Parameters (possible CPPs) for the extract production process, according to the data available in the literature for a fixed range of model proteins based on maximum reported % change in titre.

		Maximum % titre change	Average % titre change for parameter	Number of publications including pCPP	% Up vote	% Down vote
Extract	Protein tagging and removal	294	154	1	5	5
	Harvest point	100	34	5	70	
	Run‐off duration	100	100	1		5
	Run‐off use	100	65	2	15	5
	Rate of glucose feed	80	80	1	5	20
	Lysis method	80	51	4	55	
	*E*.*coli* strain	74	43	4	25	5
	Culture temperature	70	36	2		10
	Sonication burst time	42	42	1	10	10
	Sonication energy	40	40	1	10	5

*Note*: The starting or reference titre value was based on either the titre produced prior to improvement or the peak, for papers claiming to have identified a protocol improvement and papers claiming to have identified a parameter that can be optimised, respectively. The top 10 is based on the maximum absolute (i.e., whether positive or negative) values reported for % titre change. The average titre change across all studies is included for each of the top 10 possible CPPs listed. The analysis excludes articles where no titre is given (e.g., relative titres based on fluorescence or luminescence) and results where initial titres are below 50% of the average titre reported for the year of publication, implying a suboptimal system. The table includes two columns for the voting record from a workshop of CFPS active scientists, where an up‐vote indicates that the participants agreed that this was likely to be an important process parameter, and a down‐vote indicates that participants disagreed. The vote is given as a percentage of the total number of participants, where each participant had the possibility to place up to 10 up‐votes and 10 down‐votes.

**TABLE 2:** Top 10 possible Critical Process Parameters (possible CPPs) for the Cell Free Protein Synthesis reaction.

		Maximum % titre change	Average % titre change for parameter	Number of publications including pCPP	% Up vote	% Down vote
CFPS reaction	Reaction duration	220	105	5	40	
	Extract concentration	186	49	4	35	
	Reaction vessel volume:surface area	150	150	1	15	
	Temperature	148	62	5	35	
	Mg concentration	117	69	3	35	
	Mg‐glutamate concentration	100	100	1		10
	Plasmid to linear DNA	96	53	2	15	
	Energy regeneration system	88	46	8	55	
	tRNA concentration	85	85	1	5	10
	Maltose concentration	80	80	1		30

*Note*: For a detailed description, see Table 1 heading.

It is suggested that replication or further work is needed to confirm the impact of parameters that have only been studied once, which appear in the top 10 ranking when sub‐optimal/relative titres are included, or which are identified in Figures 5a and 6a as not yet covered by existing literature. As reactions in CE–CF (continuous‐exchange cell‐free) mode tend to have higher titres, and percentage changes may therefore be lower, a ranking was also conducted based on absolute titre change (data not shown), which gave a largely unchanged top 10 ranking.

#### 3.1.3 Importance of titre and robustness

As with cell‐based production, there are no generic process conditions or Critical Process Parameters (CPPs) for the myriad of different protein products which can be made using CFPS. Therefore a systematic approach is required, where the Critical Quality Attributes (CQAs) which are likely to impact the performance of a particular class of product can be linked to the possible CPPs and RMAs that are most likely to have an impact on those CQAs.

Typical CQAs for a biotherapeutic might include, but are not limited to [33, 34].

Product‐related impurities related to protein formation and chemical and physical stability (e.g., Fragmentation, truncation and aggregation)
Quality and errors in post‐translational modifications (e.g., disulphide shuffling, glycosylation pattern and deamination)
Presence of process‐related impurities (e.g., host cell proteins/DNA) and
Obligatory CQAs related to efficacy and safety (e.g., active product concentration, endotoxin and *mycoplasma* levels).

It is possible to say something about the reactions that occur in CFPS, which might impact upon CQAs. The non‐specific breakdown of energy source molecules and resultant generation of inhibitory inorganic phosphates by undesired enzymes that would be challenging to identify and remove, for example, has been identified as a key limitation to the final product titre [35]. However, current understanding within the CFPS field does not allow us to make a direct connection between all possible CPPs and CQAs of the final product.

In this article, we focus therefore on identifying possible CPPs and, where there is sufficient data/evidence in the literature, ranking them for their impact on product titre. The authors exemplify the application of systematic QbD‐inspired approaches to characterise the impact of the process on an exemplar CQA, in this case the titre, via this dataset. The logic of this focus on titre is that we need a stable, robust platform before we focus on QbD. With the increasing knowledge of the system, we expect to be able to narrow down from a large list of potential CPPs, allowing us to apply a QbD framework when looking at a new product. In any case, a reasonable titre is required before tuning to the exact system and then scaling‐up.

### 3.2 Deep dive 1: The extract production process

#### 3.2.1 Overview: Centrally produced extract as an undefined reaction raw material

Whilst with the production of a small number of centrally produced specialised protein products, it is feasible to correct for extract variability by the tweaking of the CFPS reaction, and with multiple products, this cannot easily be done for every extract batch and for every product. It is unlikely that simply running a CFPS reaction for one or more model proteins will be sufficient as a validation and control strategy for a centrally produced lysate which may be used in practice to make any number of therapeutic proteins at different distributed locations, possibly following different, protein‐specific, protocols. Extract batch‐to‐batch variability therefore needs to be monitored and controlled.

**FIGURE 8:**
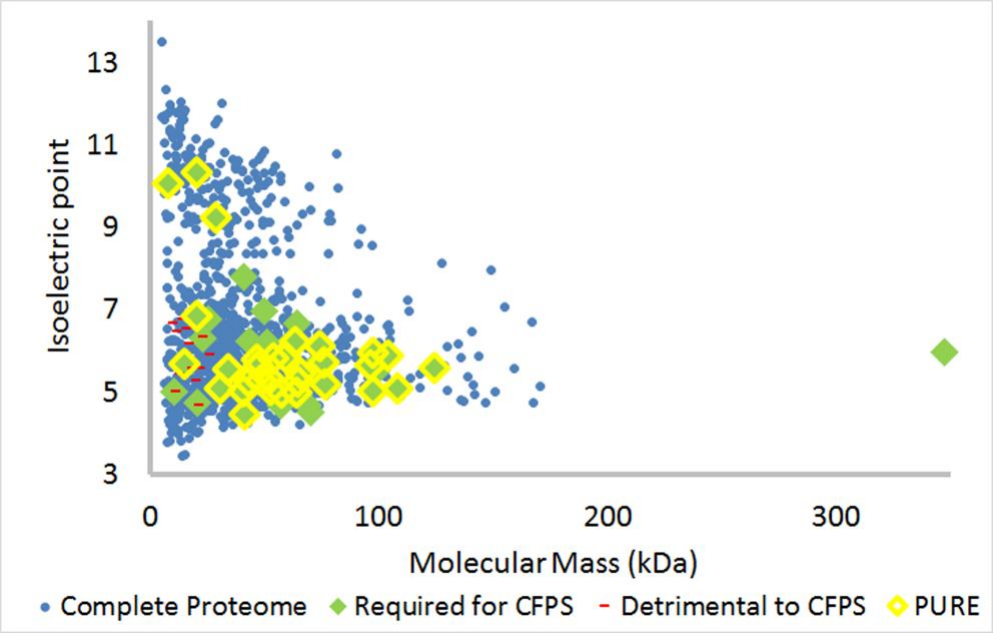
Isoelectric point versus molecular mass for the *E*. *coli* proteome, based on data reported by Ishihama et al [68] in supplementary data. A number of detrimental and beneficial proteins (including the 20 or so present in the PUrified Recombinant Elements system) are highlighted in red and green, respectively.

An *E*. *coli* cell proteome contains thousands of individual proteins (Figure 8) of which approximately 20 may be considered demonstrably essential for CFPS on the basis of their use in the PURE system. The performance of the extract is, however, likely to be impacted by the concentration and activity of these enzymes/co‐factors/chaperones as well as any number of others. The most challenging problem to overcome is therefore likely to be the measurement of batch‐to‐batch variability of the whole‐cell lysate‐based extract.

#### 3.2.2 Top 10 possible CPPs for titre control and maximisation through modification to the extract production process

For **extract production**, the harvest point [36, 37, 38, 39, 40, 41, 42], the use and duration of the run‐off reaction [37, 43, 44, 45], lysis method [37, 39, 45, 46, 47, 48] and *E*. *coli* strain [17, 42, 49, 50] are parameters which are regularly cited, though the impact shown in the literature varies widely. The impact of the harvesting time varies from none to 400%, depending seemingly upon the product, strain and growth media used. The use and duration of the run‐off reaction has an impact varying from 9% to 100%, and both positive and negative impacts are reported, which are of similar magnitude. The impact of the run‐off step may depend upon the strain, promotor and lysate preparation temperature used. The lysis method has been shown to change the final titre negligibly or up to 80%. In practice, the lysis method used is likely to be dictated by a scale or equipment available, but based on these results, optimisation is likely to have a significant impact. Finally, the impact of strain varies from 17% to 91%, which may depend upon the energy regeneration system used.

The removal of inhibitory proteins, such as nucleases or proteases, is achieved by the manipulation of the organism to either prevent the production of the inhibitory proteins at all or to tag them for removal. The identified work by Seki et al [51] was conducted using a CE–CF system and using linear DNA as a template. PCR‐generated or open‐ended linear DNA is more fragile and generally produces a lower titre, but it produces a higher baseline titre here due to use of the CE–CF reactor set‐up. It might be inferred that this single result is not therefore representative of the impact of this parameter. However, a high impact is also seen from the use of *E*. *coli* strains which omit certain proteases and RNAses [17]. In addition, some eukaryotic systems, such as the ALiCE system from LenioBio, rely heavily on the removal of certain detrimental proteins. Removing single proteins or classes of protein from a strain or species, is however, far from trivial. Work with linear DNA, where exploring this parameter may be particularly impactful, is likely to be particularly relevant to screening or prototyping applications.

The same study [52] gave a high impact for culture temperature, though this varied from 12% to 70%. Again, the significance of the reactor set‐up (CE‐CF) and use of a linear DNA template would need to be confirmed.

The glucose feed rate was identified by Zawada and Swartz [53] as being a significant factor, when defined media is used in extract production. The importance of batch‐to‐batch variability in undefined media, as a source of variability in extract properties, has not been explored in the literature. If this proved significant, then the use of defined media might be indicated. Otherwise, the importance of glucose feed in a more standard extract production protocol would need to be confirmed.

When sub‐optimal and relative titres are included, the impact on the top 10 ranked parameters is minimal for extract production, with sonication volume and lyophilisation temperature appearing in the top 10 ranking.

#### 3.2.3 Analysis and control for the extract production process

At larger scales, control of composition and concentration should become easier, whilst control of times (accounting for hold‐times and times to change temperatures) and temperature becomes more problematic. It is anticipated that extract production will be at as large a scale as feasible to benefit from economies of scale and consistent batches over a large number of CFPS reactions.

As indicated in Figure 7a, there has been some limited analysis to date of extract RMAs in the literature. The assays include measurements of the concentrations of a small number of enzymes, activity of a number of enzymes—including RNases, DNases, phosphatase and ribosomes—phospholipid and vesicle content and analysis of metabolite concentrations during a CFPS reaction. These analyses are likely to produce valuable data. However, measurement and/or reporting of these parameters is rare and generally restricted to a small number of research groups. Not enough information is therefore currently available to suggest which, if any, of these approaches should form part of the standard extract validation testing. Each of these tests is also narrow in scope and time‐consuming.

Below are some suggested approaches to cell lysate quality evaluation, which might feasibly be conducted in a semi‐automated fashion, using multi‐well plate formats, giving a broad range of data quickly.

A test of enzyme activity is required. Certain individual enzymes of particular importance can be purified and their activity and concentrations tested. A rapid approach, by contrast, might involve the use of exogenously added enzymes/substrates to saturation levels, to test the activity of a remaining enzyme, or to confirm if excess leads to generation of unwanted by‐products. This latter option would allow us to take advantage of the speed of the CFPS reaction and high‐throughput formats in which it can be run and should provide more information than simply running a CFPS reaction with unmodified extract.
Multi‐modal separation by size, charge and/or hydrophobicity followed by spectral measurements. Based on Figure 8, it would not be possible to separate all individual *E.* c*oli* proteins or even to separate essential proteins from detrimental ones. But a degree of separation should be achievable, which might allow a fingerprinting of the concentration of groups of proteins in an extract batch.

Finally, it may be worth exploring the possibility of engineering a cell‐line to be more robust to the parameters which have the biggest impact; those identified in Tables 1 and 2 may be a suitable starting point. An additional control measure which can be applied would be to pool, mix and aliquot extracts produced from multiple batches to correct for slight genetic drifts.

### 3.3 Deep dive 2: The cell‐free protein synthesis reaction

#### 3.3.1 Overview: Distributed reactions for the automated and consistent parallel production of multiple products

Once a suitably effective production protocol for a given protein has been established, assuming the batch‐to‐batch variability of the extract can be controlled, the CFPS reaction should theoretically be reproducible under these same controlled conditions.

In general, there appear to be more parameters which have been identified as having a substantial impact on titres at the CFPS reaction stage than at the extract production stage. This would appear to validate CPI's approach, at least with respect to the centralised production of a single or small number of specialised product(s) using CFPS. It is also a reflection of the greater ease with which parameters can be manipulated in the CFPS reaction, the number of reactions which can be run in parallel, the small scale at which such tests can easily be run and the speed of the reaction. In addition, many of these parameters, such as duration, temperature, extract concentration etc. can be easily monitored and controlled particularly at small scale.

#### 3.3.2 Top 10 possible CPPs for titre control and maximisation through modification of the cell‐free protein synthesis reaction

For the **CFPS reaction,** extract concentration [17, 41, 46, 54], temperature [17, 41, 55, 56], magnesium ion concentration [50, 57, 58] and the energy regeneration system [17, 49, 59, 60, 61, 62, 63, 64, 65] (i.e., supplement mixture composition) are parameters which are regularly cited, though the impact can vary widely according to the literature. The impact of extract concentration varies from 1.47%/(%v/v) up to 11.6%/(%v/v), with most showing an impact below 4%/(%v/v), with dependence on NTP concentration and use of CE–CF versus batch indicated. Temperature may impact the titre by between 4.3% and 14.8%/°C, with some indication of an impact also on solubility and activity of the product and some possible interaction with reaction duration. Magnesium ion concentration is reported as impacting titres by a fairly consistent 2.2%–3.2%/mM, with a much higher impact seen when using the PURE system [58] and with regular additions over the course of the reaction [57] (the result given in Table 2). Finally, altering the energy regeneration systems, where researchers have sought both to extend active reaction times and reduce costs associated with the supplement mixture, has resulted in improvements to titre between 14% and 197%.

The impact of reaction duration can be derived from a number of articles [17, 41, 46, 53], vary with time, particularly for batch reactions, and be dependent upon reaction set‐up (i.e., batch vs. CE‐CF). For CE–CF reactions, the titre changed by 5.9% to 11.5%/hr [46], with the extended reaction times resulting in a potentially substantial overall titre increase. By contrast, for batch reactions, the change varies from 0%/hr (after reaction completion at typically about 4 h) to 63%–133%/h [17, 41, 53] at the peak reaction rate.

Reactor format is not explored substantially, but one result from Voloshin and Swartz [66] implies that the reactor surface area to volume ratio may be significant, while substantial difference is also seen between batch and CE–CF reactions. Reactor design is an under explored area of CFPS, though practical considerations, including the simplicity of set‐up and control, robustness, scalability and reaction time lines which will also be important factors for manufacture.

When sub‐optimal and relative titres are included, the impact on the top 10 possible CPPs for the CFPS reaction is more significant than for the extract. With addition of ds DNA binding protein or elongation factor, modification of the first nucleotides of mRNA, ribosomal binding site strength, use of CE‐CF, linear DNA protection, pDNA concentration and T7 tail length all in the top 10 before these results were excluded.

#### 3.3.3 Analysis and control for the cell‐free protein synthesis reaction

At smaller scales, control of composition and concentration will become more problematic, whilst control of times (accounting for hold‐times and times to change temperatures) and temperature becomes easier. The CFPS reaction, by contrast to extract production, if applied to personalised medicine, will be relatively modest in scale.

If reagents can be prepared at large scale, mixed sufficiently for homogeneity and aliquoted, then some of the composition and concentration control issues can be mitigated. A system would need to be designed or selected that allowed important parameters, such as pH, to be sufficiently controlled. If reproducibility can be demonstrated and sufficiently validated, then monitoring of these process conditions and evaluation of the impact of any deviations should go a long way towards guaranteeing the quality of the product.

## 4 GUIDE TO FUTURE IMPROVEMENTS

### 4.1 The current state of knowledge

A limited number of examples are available of Cell‐Free Protein Synthesis (CFPS) being used or considered for the manufacture of a therapeutic drug, most notably Sutro Biopharma, Vaxcyte and Ipsen Biopharma. This paper describes the systematic approach followed by CPI, informed by experience in process development for protein production processes, for the development of a seemly robust cell‐free extract production process. Further, CPI demonstrates how, for the production of small number of products, the onus can be placed on the optimisation of the CFPS reaction, through high‐throughput multi‐well plate methodologies, where scalability is shown, with certain key environmental conditions (pH, Dissolved Oxygen, etc.) adequately controlled to a consistent level. The use case for CFPS in protein production at a large scale is limited to cases where the technology offers specific advantages.

This paper demonstrates, however, that considerable further work is required before CFPS can be treated as a viable process option for a distributed manufacture. Given the complexity of this undertaking, it may be that the first products which can be produced using CFPS as a platform process will be platform products, such as carrier proteins with small personalised structural variations, mosaic VLPs, particularly where antigen changes are required annually [7], or creation of enzymes for synthetic applications such as T7 RNA polymerase variants for mRNA. Indeed, lessons can be drawn from the spectacular progress seen in the mRNA field since the COVID pandemic and the breaking of siloes that this emergency encourage. A major factor limiting progress in CFPS is the lack of technology transfer and knowledge‐sharing efforts between labs.

Limitations in the data/literature currently available, which would need to be overcome, include

The relative dearth of data for the extract production process.
Minimal discussion of the dynamics of the CFPS reaction and changes to the limiting reaction steps and relative importance of possible CPPs.
Minimal measurement of other Critical Quality Attributes than titre, for example, Post Translation Modifications, solubility, activity, shelf‐life, extract re‐usability etc.

### 4.2 Some strategies to move the technology forward

Design of Experiment design space studies can be devised based upon the highest risk parameters identified in Tables 1 and 2, or others as needed for a given process/product. Colant et al [17] propose a rational approach which can be used for titre improvement and which can similarly be applied to robustness and to improvement of other quantifiable CQAs.

As discussed by Duran Vilalobus et al [67], the parameter set points may need to be altered over time for the best results. This is to be expected as different reaction steps and their associated extract enzymes or substrates become limiting. Identifying these dynamics may also help remove an additional source of variability in the CQAs, but literature data on this is limited to date.

Process conditions may effect not just enzymes, but also DNA and mRNA structures, impacting in turn upon their vulnerability to damage and degradation and their processibility. Some limited work has been done upon sequence modifications (linked to the initiation of enzyme interaction), with results, which require validation, indicating a potentially large impact upon product titre. In silico design may be of value in predicting the formation of higher‐dimensional structures from DNA and mRNA sequences and their interactions with the relevant enzymes, and the impact of process parameters, particularly pH and salt concentration, on these.

The dearth of data for the extract production process could be best address by the development of scale‐down methods designed to imitate large‐scale production, and building of mechanistic understanding and experimentally validated simulations. The challenges of scaling‐down extract production for possible Critical Process Parameter analysis are likely to centre around lysis by homogenisation, which will limit the minimum possible scale.

Broader process considerations will also need to be taken into account. These include, but are likely not limited to

Simplicity of protocol versus operating window size, that is, the risk of operator error versus process variability
Having the option to use linear DNA templates, instead of plasmids, for the ease of production
Scalability and use of process units relevant to industrial scale production.
Conditions which minimise the risk of evolution/genetic variation being generated in the cells
Incorporating manufacturability into the screening process using CFPS [34].

### 4.3 The long‐term vision

Whilst it is unlikely to replace existing biomolecule production methodologies, CFPS offers substantial potential in a number of fields. This includes for the production of personalised medicines, which is of particular interest to researchers on the Future Targeted Healthcare Manufacturing hub at UCL. It is envisioned that through the use of large‐scale, robust and central cell‐free extract production and high‐throughput process development for the CFPS reactions, a range of personalised products can be produced at appropriate scales, using simple reaction systems, close to the point of use.

**FIGURE 9:**
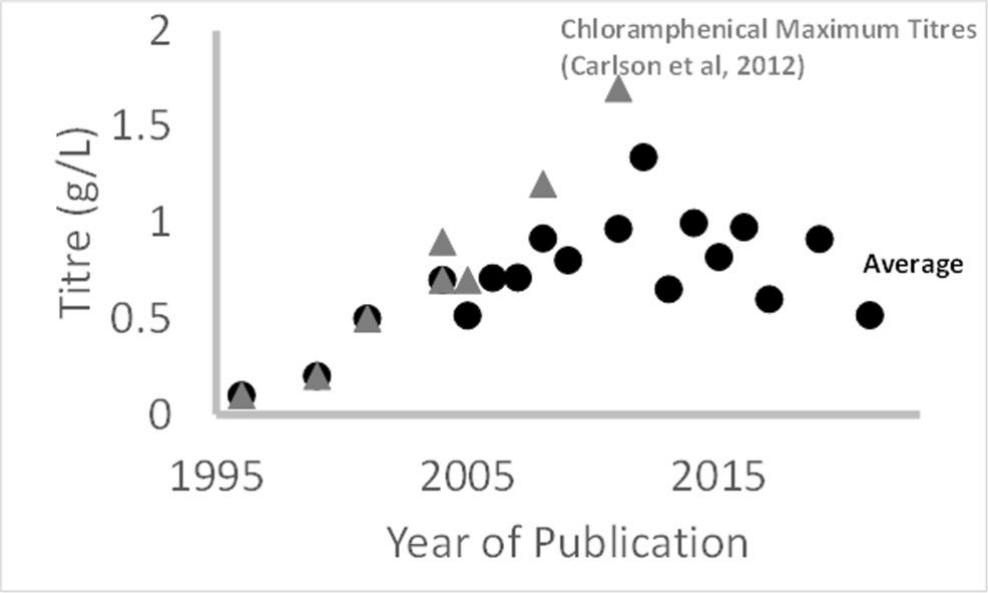
Trend of reported titres of model proteins against publication year, showing year average across a range of model proteins (CAT, GFPs, gluc) (circles) and maximum titres of CAT as reported by Carlson et al, 2012 (triangles).

This potential is limited by the current underperformance of the system. Moreover, good practice is poorly shared across the field resulting in a lack of consistent improvements in titre over the last 2 decades (Figure 9). In this article, the authors have attempted to draw lessons towards development of a systematic and robust framework for CFPS process development. It is hoped that lessons drawn from this analysis will serve to focus analytic choices and accelerate titre improvements—a first step towards building understanding and development of robust and viable CFPS production processes.

## 5 CONCLUSIONS

CPI has demonstrated a logical approach to the development of a cell‐extract production process and high‐throughput approaches to the optimisation of the subsequent CFPS reaction, within the constraints of commercial budget and timelines. The challenge for the research and industrial communities, with an interest in CFPS going forwards, will be the systematisation of their approach to process improvement and reporting. In this article, the authors have endeavoured to draw together evidence from various sources as an initial guide to focus such a systematisation.

## AUTHOR CONTRIBUTIONS

**Beatrice Judith Melinek**: Conceptualisation; data curation; formal analysis; methodology; visualisation; writing – original draft; writing – review & editing. **Jade Tuck**: Conceptualisation; data curation; methodology; writing – original draft; writing – review & editing. **Philip Probert**: Conceptualization; methodology; supervision; writing – review & editing. **Harvey Branton**: Conceptualisation; methodology; supervision; writing – review & editing. **Daniel G. Bracewell**: Conceptualisation; supervision; writing – review & editing.

## CONFLICT OF INTEREST STATEMENT

The authors declare no conflicts of interest.

## Supporting information

Supporting Information S1

## Data Availability

Data sharing is not applicable to section 3 of this article as no new data were created or analysed in this study. Research data are not shared for section 2 of this article.
