# Supplementary material for: Designing of an extract production protocol for industrial application of cell‐free protein synthesis technology: Building from a current best practice to a quality by design approach
Source: Eng Biol. 2023 Dec 6;7(1-4):1–17. doi: 10.1049/enb2.12029 (PMC10715128; doi:10.1049/enb2.12029)
Supplement: Supplementary file 1 — Supporting Information S1 [file ENB2-7-1-s001.docx]

Supplementary data:

Data for minimal media

Full literature search for extract production

Full literature search for CFPS reaction
